# Supplementary material for: C5aR1 signaling promotes region‐ and age‐dependent synaptic pruning in models of Alzheimer's disease
Source: Alzheimers Dement. 2024 Jan 26;20(3):2173–90. doi: 10.1002/alz.13682 (PMC10984438; doi:10.1002/alz.13682)
Supplement: Supplementary file 1 — Supporting Information [file ALZ-20-2173-s004.pdf]

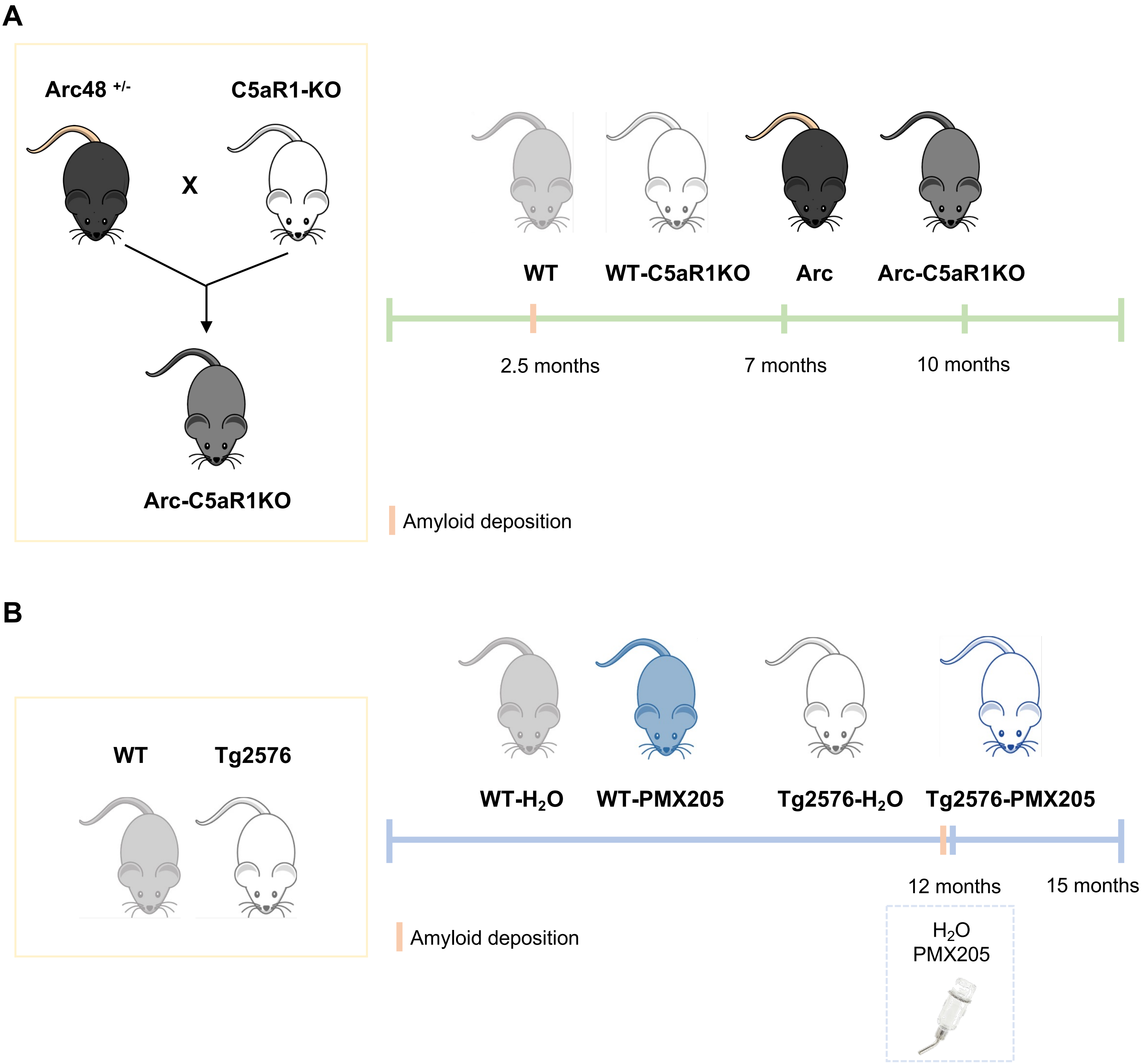

Supplemental Figure 1: Experimental design.

A. Schematic diagram of the experimental design showing that Arc48+/- mice were crossed with C5aR1-KO mice to create Arctic mice lacking C5aR1 (Arc-C5aR1KO). For the experiments shown in this manuscript, WT, WT-C5aR1KO, Arc and Arc-C5aR1KO mice were aged to 2.7, 5, 7 and 10 months of age. Amyloid pathology is prominent in this mouse model at 4 mo of age. **B**. Schematic diagram of the experimental design showing that Tg2576 mice and WT littermates were treated with 20 µg/ml of PMX205 in drinking water (or H_2O) for 12 weeks from 12-15 months of age, the time corresponding to the onset and accumulation of amyloid pathology in this mouse model of AD.
