# Supplementary material for: C5aR1 signaling promotes region‐ and age‐dependent synaptic pruning in models of Alzheimer's disease
Source: Alzheimers Dement. 2024 Jan 26;20(3):2173–90. doi: 10.1002/alz.13682 (PMC10984438; doi:10.1002/alz.13682)
Supplement: Supplementary file 2 — Supporting Information [file ALZ-20-2173-s005.pdf]

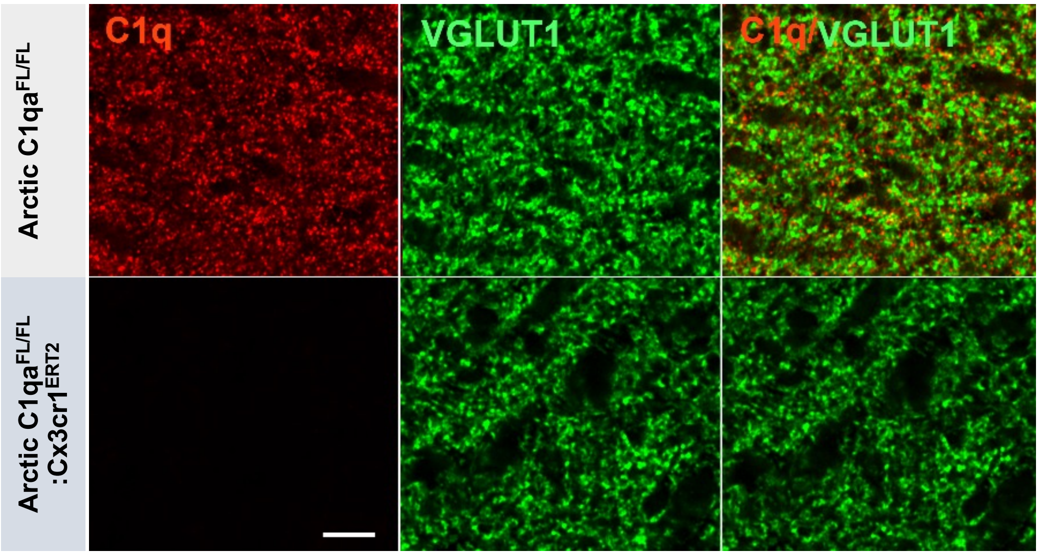

Supplemental Figure 2: Specificity of rabbit monoclonal anti-mouse C1q Representative super-resolution images of VGlut1 (green), C1q (red) and merged C1q-VGlut1 in the CA1-SR hippocampal region of Arctic-C1qa$^{FL/FL}$ (top panel) and C1qa deleted Arctic-C1qa$^{FL/FL}$:Cx3cr1^{ERT2} (bottom panel) at 10 months of age. Scale bar: 5 μm.
