# Supplementary material for: C5aR1 signaling promotes region‐ and age‐dependent synaptic pruning in models of Alzheimer's disease
Source: Alzheimers Dement. 2024 Jan 26;20(3):2173–90. doi: 10.1002/alz.13682 (PMC10984438; doi:10.1002/alz.13682)
Supplement: Supplementary file 3 — Supporting Information [file ALZ-20-2173-s001.pdf]

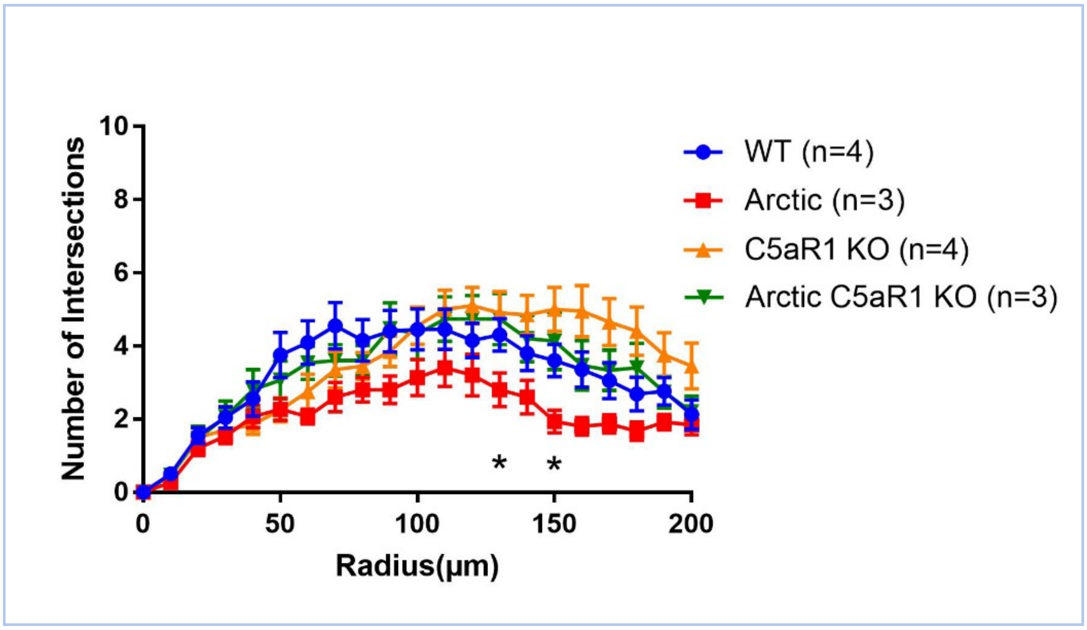

Supplemental Figure 3: Loss of neuronal complexity in CA3 is prevented by C5aR1 ablation in the Arctic model of AD at 10 months.

Sholl analysis of WT, Arctic, C5aR1-KO, and Arc-C5aR1KO in the CA3 area at 10 months of age. Neurite length intersections between 0 and 200 μm from the soma were averaged in 10-20 neurons per mouse from 3-4 mice per genotype. Data are shown as Mean ± SEM. *$p<0.05$ when comparing Arc vs Arc-C5aR1KO. In addition, using Kolmogorov-Smirnov test, WT vs Arctic, $p=0.006$; Arc vs Arc-C5aR1KO, $p=0.002$; whereas WT vs Arc-C5aR1KO is not significantly different.
