# Supplementary material for: C5aR1 signaling promotes region‐ and age‐dependent synaptic pruning in models of Alzheimer's disease
Source: Alzheimers Dement. 2024 Jan 26;20(3):2173–90. doi: 10.1002/alz.13682 (PMC10984438; doi:10.1002/alz.13682)
Supplement: Supplementary file 4 — Supporting Information [file ALZ-20-2173-s002.pdf]

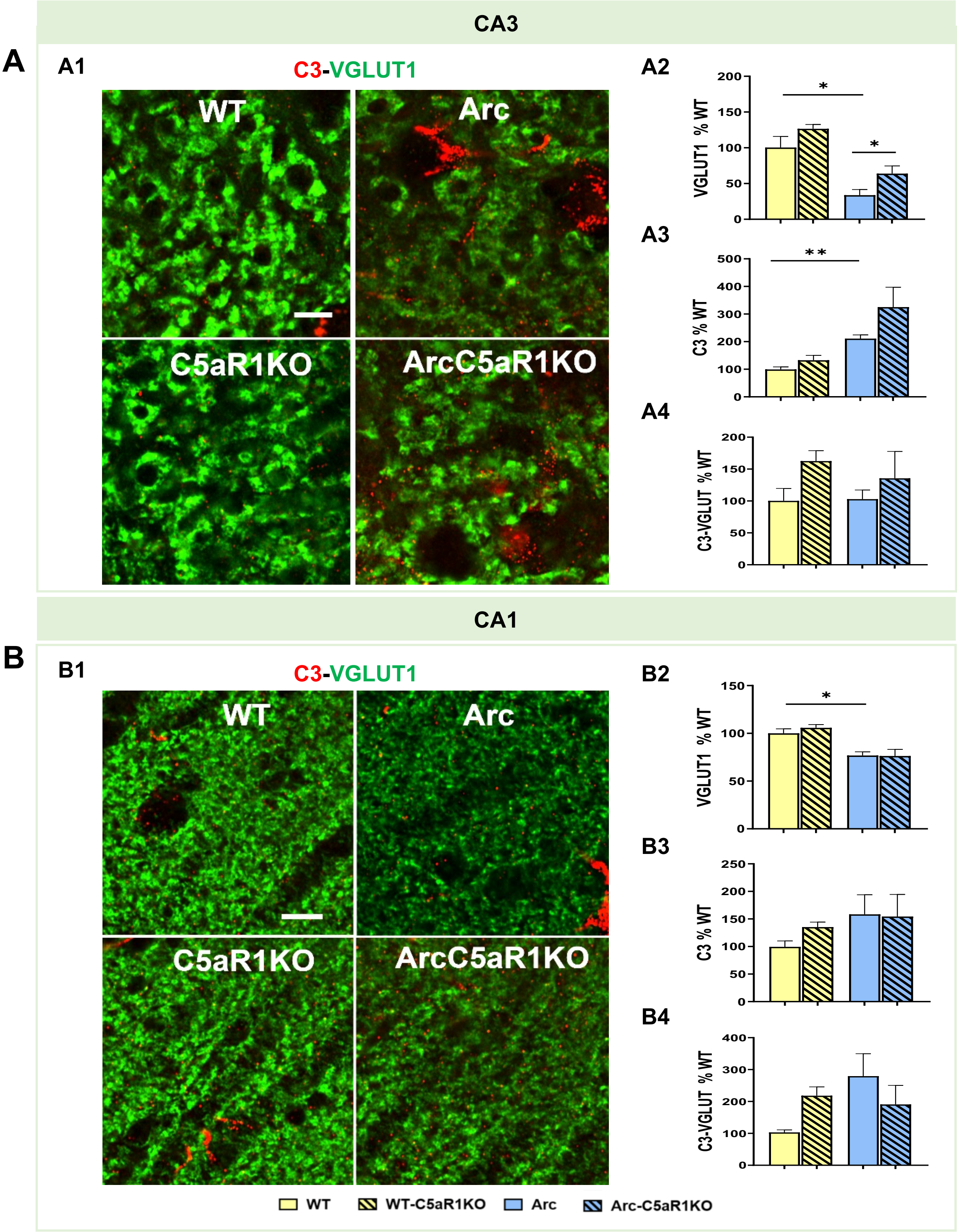

Supplemental Figure 4: C3 tagging of VGlut1 presynaptic puncta at 10 months of age.

A. Representative super-resolution images (A1) and quantitative analysis of C3, VGlut1 and C3-VGlut1 colocalized puncta (A2-A4) in the CA3-SL region. **B**. Representative super-resolution images (B1) and quantitative analysis of C3, VGlut1 and C3-VGlut1 colocalized puncta (B2-B4) in the CA1-SR region. Scale bar: 5 um. Data are shown as Mean ± SEM (normalized to WT control group) of 3 images per animal and n=3-4 animals per genotype. *p<0.05; **p<0.01 using one-way ANOVA followed by Tukey's post hoc test (A-B) or unpaired t test (A).
