# Supplementary material for: C5aR1 signaling promotes region‐ and age‐dependent synaptic pruning in models of Alzheimer's disease
Source: Alzheimers Dement. 2024 Jan 26;20(3):2173–90. doi: 10.1002/alz.13682 (PMC10984438; doi:10.1002/alz.13682)
Supplement: Supplementary file 5 — Supporting Information [file ALZ-20-2173-s007.pdf]

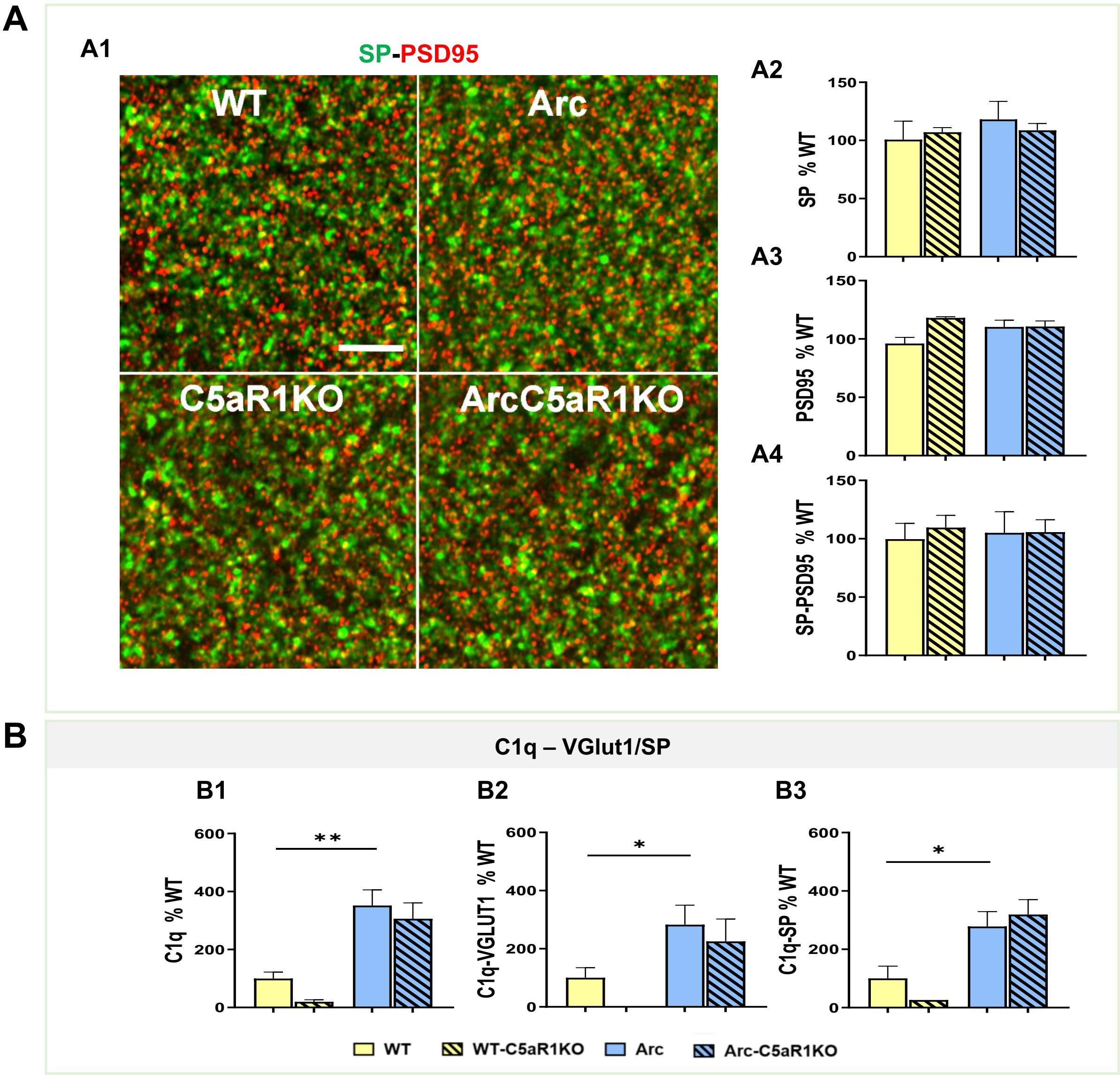

Supplemental Figure 5: No differences in synaptic density despite an increase in C1q tagging of presynaptic puncta in the DG-ML at 10 months of age.

A. Super-resolution images of SP (green) and PSD95 (red) in the DG-ML of 10m Arc, Arc-C5aR1KO and their respective WT littermates. Scale bar: 5 µm (A1). Imaris quantification of SP, PSD95 and SP-PSD95 co-localized puncta (A2-A4). Data are shown as Mean ± SEM (normalized to WT control group) of 3 images per animal and n=2-3 animals per genotype. **B**. Quantitative analysis of C1q, C1q-VGLUT1 and C1q-SP colocalized puncta. Data are shown as Mean ± SEM (normalized to WT control group) of 3 images per animal and n=1-4 (C1q-SP) or n=3-7 (C1q-VGLUT1) mice per group. * $p<0.05$; ** $p<0.01$ using one-way ANOVA followed by Tukey's post hoc test (B).
