# Supplementary material for: C5aR1 signaling promotes region‐ and age‐dependent synaptic pruning in models of Alzheimer's disease
Source: Alzheimers Dement. 2024 Jan 26;20(3):2173–90. doi: 10.1002/alz.13682 (PMC10984438; doi:10.1002/alz.13682)
Supplement: Supplementary file 6 — Supporting Information [file ALZ-20-2173-s008.pdf]

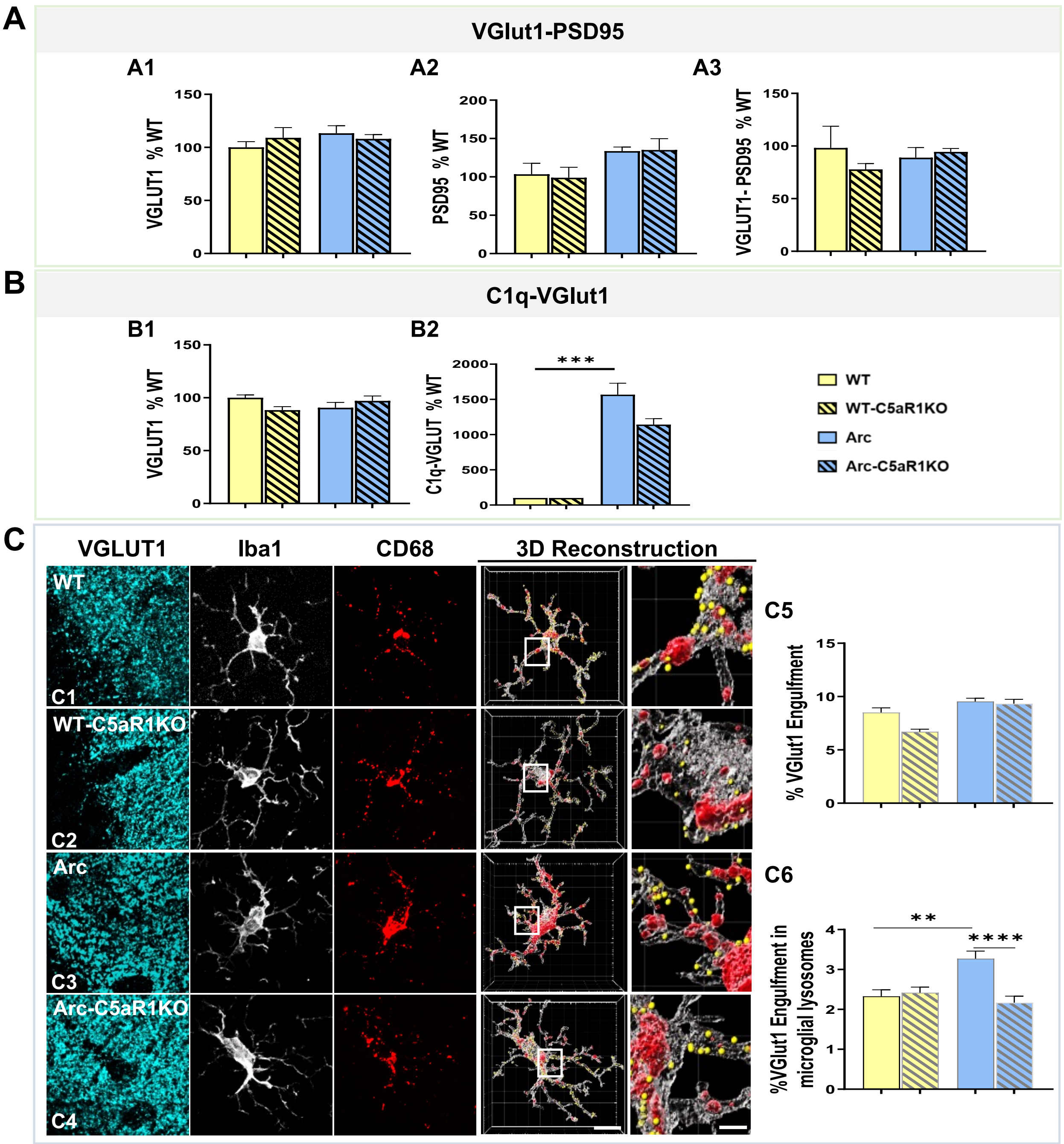

Supplemental Figure 6: VGlut1 microglial engulfment or synaptic density remain unchanged despite an excessive C1q tagging in the CA1-SR hippocampal region at 7 months in the Arctic model of AD. Quantification of super-resolution images of (**A**) VGlut1, PSD95 and colocalized VGlut1-PSD95 puncta or (**B**) of VGlut1 and C1q-VGlut1 puncta in CA1-SR at 7 months of age. Data are shown as Mean ± SEM (normalized to WT control group) of 3 images per mouse and n=3-4 mice per genotype. **C**. Confocal images and 3D surface rendering of Iba1, CD68 and VGlut1 engulfment at 7 months of age in the CA1-SR region of the hippocampus. Scale bar: 10 µm; inserts 2 µm (C1-C4). Quantitative analysis of VGlut1+ presynaptic puncta engulfment per microglia (C5) or localized within the microglial lysosomes (C6). Data are shown as Mean ± SEM of 15 individual microglial cells/mouse and n=3-4 mice per genotype. *p<0.05, ** p<0.01, *** p<0.001, **** p<0.0001 using one-way ANOVA followed by Tukey's post hoc test (A-C).
