# Supplementary material for: C5aR1 signaling promotes region‐ and age‐dependent synaptic pruning in models of Alzheimer's disease
Source: Alzheimers Dement. 2024 Jan 26;20(3):2173–90. doi: 10.1002/alz.13682 (PMC10984438; doi:10.1002/alz.13682)
Supplement: Supplementary file 7 — Supporting Information [file ALZ-20-2173-s006.pdf]

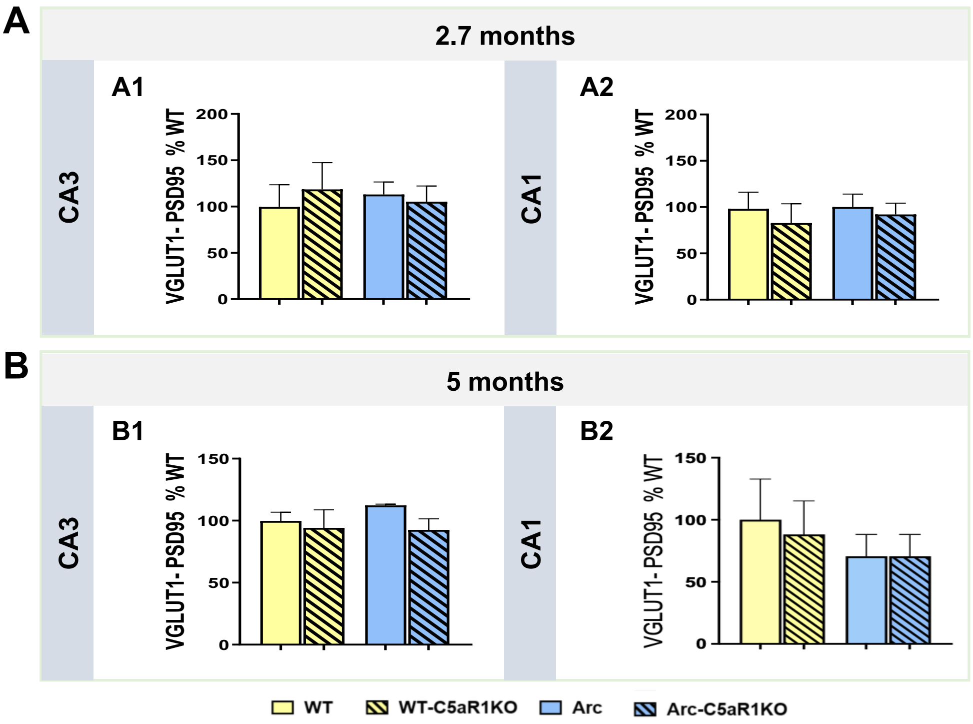

Supplemental Figure 7: No differences in synaptic density were observed in CA3-SL or CA1-SR at 2.7 or 5 months of age.

A-B. Quantitative analysis of colocalized VGlut1-PSD95 puncta at 2.7 months (A) or 5 months (B) of age in the CA3-SL (A1, B1) and CA1-SR (A2, B2) hippocampal regions. Data are shown as Mean ± SEM (normalized to WT control group) of 3 images per animal and n=3 animals per genotype.
